# A decreased abundance of clostridia characterizes the gut microbiota in eosinophilic esophagitis

**DOI:** 10.14814/phy2.14261

**Published:** 2019-10-24

**Authors:** Purna C. Kashyap, Stephen Johnson, Debra M. Geno, Heather R. Lekatz, Crystal Lavey, Jeffrey A. Alexander, Jun Chen, David A. Katzka

**Affiliations:** ^1^ Gastroenterology and Hepatology Mayo Clinic Rochester Minnesota; ^2^ Physiology and Biomedical Engineering Mayo Clinic Rochester Minnesota; ^3^ Biomedical Statistics and Informatics Mayo Clinic Rochester Minnesota

**Keywords:** Microbiome, eosinophilic esophagitis, allergy

## Abstract

Abnormalities in the gut microbiome are associated with suppressed Th2 response (Belizario et al., 2018 *Mediators Inflamm*. 2018:2037838) and predisposition to atopic disease such as asthma and eczema. We investigated if this applies to eosinophilic esophagitis (EoE). Stool bacterial DNA was extracted and followed by 16S rRNA amplification from 12 patients with eosinophilic esophagitis and 12 controls. Alpha‐ and beta‐diversity were analyzed. Only two patients had asthma or atopy and one patient was on budesonide. No patients were on PPIs. Patients with EoE had lower gut microbiota alpha diversity (species richness, *P = 0.09*; Shannon index, *P* = 0.01). The microbial composition was distinct as evidenced by significantly different beta diversity (*P* = 0.03) when compared to healthy controls. There were also significant differences in relative abundance at multiple taxonomic levels when comparing the two communities; at the phylum level, we observed a marked decrease in Firmicutes and increase in Bacteroidetes and at the order and family level there were significant decreases in *Clostridia* and *Clostridiales* in patients with EoE (*q* ≤ 0.1). We conclude that there are significant differences in microbial community structure, microbial richness, and evenness and a significant decrease in taxa within the *Clostridia* in patients with EoE. Our data suggest that *Clostridia* based interventions could be tested as adjuncts to current therapeutic strategies in EoE.

## Introduction

Abnormalities in the gut microbiome are associated with perturbations in systemic immune reaction (Belizario et al., 2018) and predisposition to atopic disease such as asthma and eczema (Zimmermann et al., 2019). While several genetic (Sherrill and Rothenberg, 2011), environmental (Hill and Spergel, 2018) and esophageal microbiome (Benitez et al., 2015; Harris et al., 2015; Norder Grusell et al., 2018) factors have been implicated in EoE, there is evidence that supports a role for the gut microbiome. For example, factors associated with altered gut microbiome such as lack of breast feeding and performance of Cesarean section(Jensen et al., 2018) or early life antibiotic use (Radano et al., 2014) have been associated with EoE. Multiple studies in human subjects have found an association between altered gut microbiota and atopic disorders (Nimwegen et al., 2011; Ismail et al., 2012; Lau et al., 2012) and impaired mucosal barrier function (Katzka et al., 2014; Sherrill et al., 2014) in response to circulating cytokines (Sherrill et al., 2014). The sensitization to food antigens triggering an immune response and driving esophageal inflammation in EoE (Noti et al., (2013)) also suggests a role for gut microbes as commensal bacteria within C*lostridium* clusters XIVa, XIVb, and IV have previously been described to protect against development of food allergies in rodent studies by driving expansion of FoxP3 + regulatory T cells (Tregs) and improving barrier function (Stefka et al., 2014). Finally the positive response to elemental diet seen in EoE (Kelly et al., 1995; Markowitz et al., 2003; Warners et al., 2017) with its potential effects on stool microbiota{Kajiura, 2009 #17869}points to role for gut microbiome. Hence in this study we evaluated the gut microbiome represented in the stool from patients with EoE and compared it with matched healthy controls.

## Methods

### Ethical approval of human studies

All human studies were approved by the Mayo Clinic IRB [EoE (16‐001097), healthy controls (14‐003005, 16‐006388)].

### Patients

Adult patients with eosinophilic esophagitis defined by consensus criteria (Dellon et al., 2013) were sequentially recruited from our eosinophilic esophagitis outpatient clinic. Patients were excluded if they were on proton pump inhibitors, had antibiotic exposure or systemic steroids within the past 2 months. Patients mailed the stool sample in a cold pack within 1 week of endoscopy which was then stored in −80°C till further processing. Healthy adult control patients were recruited as part of different studies and matched with the EoE patients for age, sex and BMI. None of the healthy controls had history of asthma, antibiotic use within past 4 weeks or PPI use. Only one control was delivered by C‐section and no controls had early life antibiotic exposure. All were on a regular diet.

### Microbiome analysis

Stool bacterial DNA was extracted using MoBio fecal DNA extraction kit, followed by 16S rRNA amplification using Nextera library compatible primers flanking the V4 hypervariable region ([forward overhang] + 515F: [TCGTCGGCAGCGTCAGATGTGTATAAGAGACAG]GTGCCAGCMGCCGCGGTAA; and [reverse overhang] + 806R: [GTCTCGTGGGCTCGGAGATGTGTATAAGAGACAG]GGACTACHVGGGTWTCTAAT) and prepared for sequencing using a dual‐indexing protocol. All samples were sequenced together in 2x300 paired‐end mode on an Illumina MiSeq instrument using v3 reagents by the University of Minnesota Genomics Center. Paired R1 and R2 sequence reads were processed via the *hybrid‐denovo* bioinformatics pipeline (Chen et al., 2018), which clustered good‐quality paired and single‐end reads into operational taxonomic units (OTUs) at 97% similarity level. In total, 989,199 reads (median: 41,727 reads per sample, range: 21,861 to 53,839 reads per sample) were included. OTUs were assigned taxonomy using RDP classifier trained on the Silva database (v132). These OTUs belong to 15 phyla, 84 families, and 248 genera.

Alpha‐diversity (species richness (observed number of OTU) and Shannon index) and beta‐diversity (unweighted, generalized (*α* = 0.5), and weighted UniFrac and Bray‐Curtis distance) were analyzed for the OTU data (R “GUniFrac” package v1.1; Chen et al., 2012). Rarefaction was performed on the OTU table for the diversity analyses (rarefied to 20,000 reads per sample). A linear regression model (*t*‐test) was used for testing the association with alpha‐diversity. Beta‐diversity association was tested using PERMANOVA based on the aforementioned distance measures. Taxa‐level association analyses were performed at multiple taxonomic levels. The count data were normalized by GMPR size factor to address potential compositional effects. Taxa with prevalence less than 10% or with a maximum proportion less than 0.2% were excluded. A permutation test (1000 permutations) was used to identify differentially abundant taxa based on the t‐statistic of a linear regression model with the square‐root transformed taxa relative abundance as the response variable. Permutation‐based false discovery rate (FDR) control was used to correct for multiple testing on each taxonomic level, and FDR‐adjusted p‐values or *q*‐values ≤ 0.1 were considered significant. All statistical analyses were performed in R 3.4.2.

## Results

Twelve patients with eosinophilic esophagitis (eight active) had a median age of 48 (34‐64), body mass index (BMI) was 29.51 (22.40–36.49), four were male and all were Caucasian (Table 1). Only one patient had asthma and one had atopic dermatitis. Six patients were on a regular diet. No patients were on PPIs. The healthy control patients had a median age 51 (33–61), BMI 24.75 (19.73–41.39), three were male and all were Caucasian. None had a history of asthma, atopic dermatitis, or PPI use.

**Table 1:** Clinical and demographic characteristics of study patients.

	Age	Gender	Allergies	Asthma	atopic dermatitis	Delivered by C‐section	Hx of antibiotics < 1 year old	Breast feed	Meds	EoE Diet therapy
Active EoE	34	F	Seasonal, Dogs/cats	Yes	No	Yes	No	No	albuterol	Yes
	42	M	Seasonal	No	No	No	Yes	No	Albuterol Flonase	No
	44	F	None	No	No	No	No	No	Multivitamin	No
	49	F	Seasonal	No	No	No	No	Yes	Synthroid, Lisinophril	Yes
	53	F	None	No	No	No	No	UNK	Adderall Flonase	No
	54	F	None	No	No	No	No	UNK	Norvasc Neurontin, Wellbutrin‐XL	No
	58	M	Cat dander	No	No	No	No	UNK	Afrin Lipitor	No
	64	M	MSG, bee stings	No	No	No	No	No	Flovent Zantac	No
Inactive EoE	37	F	None	No	No	No	No	Yes	Allegra, montelukast Zantac	Yes
	46	F	None	No	No	No	No	No	Zoloft	Yes
	46	F	Animals/dust	No	No	No	No	No	Budesonide	No
	52	M	Seasonal/animal	No	Yes	UNK	UNK	UNK	Sidenafil Synthroid	Yes

**Figure 1:**
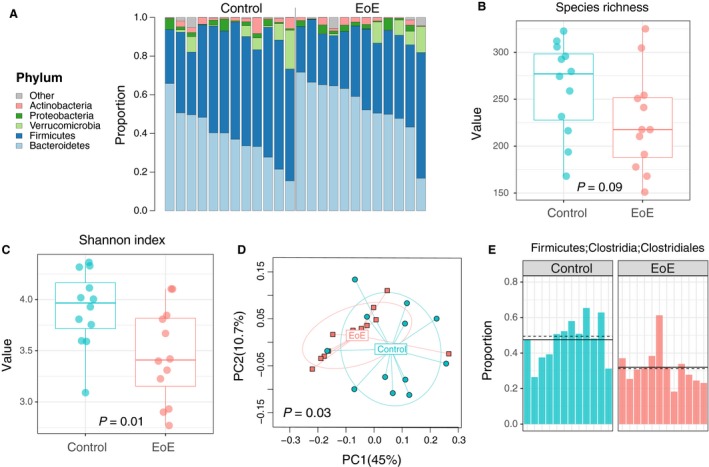
The phylum‐level profiles (A), alpha diversity (within subject) based on species richness (B) and Shannon index (C), principal coordinate analysis (PCoA) plot showing beta diversity based on weighted UniFrac (D), and barplot comparing the abundance of Clostridia/*Clostridiales* (E) among healthy controls (*n* = 12) and EoE patients (*n* = 12).

We compared the 16S rRNA‐based microbial community composition in stool samples from the patients with EoE (*n* = 12) and healthy control subjects (*n* = 12). At the phylum level, we observed a marked decrease in Firmicutes and increase in Bacteroidetes in patients with EoE (Fig. 1A). The gut microbial communities from patients with EoE were characterized by a lower alpha diversity (species richness, *P = 0.09*; Shannon index, *P* = 0.01; linear regression; Fig. 1B, 1). The beta‐diversity was also significantly different between patients with EoE and healthy controls (*P* = 0.03, PERMANOVA based on weighted UniFrac; *P* = 0.04, omninbus test combining multiple beta diversity measures; Fig. 1D). There were significant differences in relative abundance at multiple taxonomic levels when comparing the two communities, which included significant decreases in *Clostridia* and *Clostridiales* in patients with EoE (*q* ≤ 0.1, permutation test with t‐statistic; Fig. 1E**)**.

## Discussion

In our pilot study we found significant differences in microbial community structure and a decrease in microbial richness and evenness in patients with EoE. Additionally we found a significant decrease in taxa within the Clostridia in patients with EoE. In the differential abundance analysis, we used a permutation test based on the normalized and transformed data, thus, the observed difference in Clostridia was less likely to be driven by sequence depth variation and outliers. In fact, similar *P*‐values were obtained even if we rarefied the data to 20,000 reads per sample. This finding is particularly important as previous studies show that members of *Clostridia* group are protective against the development of food allergies in a rodent model. *Clostridia*‐containing microbiota was associated with the expansion of intestinal regulatory T cells (Treg), induction of IL‐22 production by RORγt^+^ ILCs and class switching to IgA promoting an immune environment conducive for tolerance to dietary antigens (Stefka et al., 2014). The abundance of *Clostridiales* correlated with the expression of antimicrobial peptide *Reg3b* suggesting *Reg3b* may play a role in modulating the abundance of Cl*ostridiales* in the colon (Stefka et al., 2014). In addition to rodent studies, *Clostridia* strains isolated from healthy human feces have also been shown to induce Tregs in colonic lamina propria when transferred to germ‐free mice (Nagano et al., 2012).

Our study is limited by the small sample size although the ability to show significant differences given the number of variables in study of the microbiome is noteworthy. Strengths of the study are a lack of medications, such as proton pump inhibitors (Bajaj et al., 2018), or other atopic illnesses in most patients that have been associated with altered stool microbiome. This is difficult to achieve as most patients with atopic illness associated with microbiome differences commonly have other atopic conditions and/or usage of medications for these diseases. Furthermore, we limited our analyses to stool samples as we were interested in the association of an altered gut microbiome rather than the esophageal microbiome as a change in the former has been reported to predispose to organ specific atopic disorders, such as asthma, later in life. On the other hand, we cannot precisely identify the relationship of early life changes in stool microbiome to what is found in these patients as adults. Finally, whether our data support the use of Clostridia based interventions as adjuncts to current therapeutic strategies in EoE needs to be further assessed.

## Conflict of Interest

Purna C Kashyap is on the advisory board of uBiome and as an ad hoc advisory board member for Salix pharmaceuticals. David A. Katzka is on the advisory board for Shire and Celgene.

## Supporting information




**Figure S1**: Proposed pathogenesis of microbiome in eosinophilic esophagitis.

## References

[phy214261-bib-0001] Bajaj, J. S. , C. Acharya , A. Fagan , M. B. White , E. Gavis , D. M. Heuman , et al. 2018 Proton pump inhibitor initiation and withdrawal affects gut microbiota and readmission risk in Cirrhosis. Am. J. Gastroenterol. 113:1177–1186. 29872220 10.1038/s41395-018-0085-9

[phy214261-bib-0002] Belizario, J. E. , J. Faintuch , and M. Garay‐Malpartida . 2018 Gut microbiome dysbiosis and immunometabolism: new frontiers for treatment of metabolic diseases. Mediators Inflamm. 2018:2037838. 30622429 10.1155/2018/2037838 PMC6304917

[phy214261-bib-0003] Benitez, A. J. , C. Hoffmann , A. B. Muir , K. K. Dods , J. M. Spergel , F. D. Bushman , et al. 2015 Inflammation‐associated microbiota in pediatric eosinophilic esophagitis. Microbiome 3:23. 26034601 10.1186/s40168-015-0085-6 PMC4450515

[phy214261-bib-0004] Chen, J. , K. Bittinger , E. S. Charlson , C. Hoffmann , J. Lewis , G. D. Wu , et al. 2012 Associating microbiome composition with environmental covariates using generalized UniFrac distances. Bioinformatics 28:2106–2113. 22711789 10.1093/bioinformatics/bts342 PMC3413390

[phy214261-bib-0005] Chen, X. , S. Johnson , P. Jeraldo , J. Wang , N. Chia , J. A. Kocher , et al. 2018 Hybrid‐denovo: a de novo OTU‐picking pipeline integrating single‐end and paired‐end 16S sequence tags. Gigascience 7:1–7. 10.1093/gigascience/gix129 PMC5841375 29267858

[phy214261-bib-0006] Dellon, E. S. , N. Gonsalves , I. Hirano , G. T. Furuta , C. A. Liacouras , D. A. Katzka . and American College of G . 2013 ACG clinical guideline: Evidenced based approach to the diagnosis and management of esophageal eosinophilia and eosinophilic esophagitis (EoE). Am. J. Gastroenterol 108:679–692; quiz 693. 23567357 10.1038/ajg.2013.71

[phy214261-bib-0007] Harris, J. K. , R. Fang , B. D. Wagner , H. N. Choe , C. J. Kelly , S. Schroeder , et al. 2015 Esophageal microbiome in eosinophilic esophagitis. PLoS ONE 10:e0128346. 26020633 10.1371/journal.pone.0128346 PMC4447451

[phy214261-bib-0008] Hill, D. A. , and J. M. Spergel . 2018 Is eosinophilic esophagitis a member of the atopic march? Ann. Allergy Asthma Immunol. 120:113–114. 29413330 10.1016/j.anai.2017.10.003 PMC8019101

[phy214261-bib-0009] Ismail, I. H. , F. Oppedisano , S. J. Joseph , R. J. Boyle , P. V. Licciardi , R. M. Robins‐Browne , et al. 2012 Reduced gut microbial diversity in early life is associated with later development of eczema but not atopy in high‐risk infants. Pediatr. Allergy Immunol. 23:674–681. 22831283 10.1111/j.1399-3038.2012.01328.x

[phy214261-bib-0010] Jensen, E. T. , J. T. Kuhl , L. J. Martin , C. D. Langefeld , E. S. Dellon , and M. E. Rothenberg . 2018 Early‐life environmental exposures interact with genetic susceptibility variants in pediatric patients with eosinophilic esophagitis. J. Allergy Clin. Immunol. 141:632–637.e5. 29029802 10.1016/j.jaci.2017.07.010 PMC5803324

[phy214261-bib-0011] Katzka, D. A. , R. Tadi , T. C. Smyrk , E. Katarya , A. Sharma , D. M. Geno , et al. 2014 Effects of topical steroids on tight junction proteins and spongiosis in esophageal epithelia of patients with eosinophilic esophagitis. Clin. Gastroenterol. Hepatol. 12:1824–1829.e1. 24681080 10.1016/j.cgh.2014.02.039

[phy214261-bib-0012] Kelly, K. J. , A. J. Lazenby , P. C. Rowe , J. H. Yardley , J. A. Perman , and H. A. Sampson . 1995 Eosinophilic esophagitis attributed to gastroesophageal reflux: improvement with an amino acid‐based formula. Gastroenterology 109:1503–1512. 7557132 10.1016/0016-5085(95)90637-1

[phy214261-bib-0013] Lau, S. , K. Gerhold , K. Zimmermann , C. W. Ockeloen , S. Rossberg , P. Wagner , et al. 2012 Oral application of bacterial lysate in infancy decreases the risk of atopic dermatitis in children with 1 atopic parent in a randomized, placebo‐controlled trial. J. Allergy Clin. Immunol. 129:1040–1047. 22464674 10.1016/j.jaci.2012.02.005

[phy214261-bib-0014] Markowitz, J. E. , J. M. Spergel , E. Ruchelli , and C. A. Liacouras . 2003 Elemental diet is an effective treatment for eosinophilic esophagitis in children and adolescents. Am. J. Gastroenterol. 98:777–782. 12738455 10.1111/j.1572-0241.2003.07390.x

[phy214261-bib-0015] Nagano, Y. , K. Itoh , and K. Honda . 2012 The induction of Treg cells by gut‐indigenous Clostridium. Curr. Opin. Immunol. 24:392–397. 22673877 10.1016/j.coi.2012.05.007

[phy214261-bib-0016] van Nimwegen, F. A. , J. Penders , E. E. Stobberingh , D. S. Postma , G. H. Koppelman , M. Kerkhof , et al. 2011 Mode and place of delivery, gastrointestinal microbiota, and their influence on asthma and atopy. J. Allergy Clin. Immunol. 128:e941–943. 10.1016/j.jaci.2011.07.027 21872915

[phy214261-bib-0017] Norder Grusell, E. , G. Dahlen , M. Ruth , H. Bergquist , and M. Bove . 2018 The cultivable bacterial flora of the esophagus in subjects with esophagitis. Scand. J. Gastroenterol. 53:650–656. 29616839 10.1080/00365521.2018.1457712

[phy214261-bib-0018] Noti, M. , E. D. Wojno , B. S. Kim , M. C. Siracusa , P. R. Giacomin , M. G. Nair , et al. 2013 Thymic stromal lymphopoietin‐elicited basophil responses promote eosinophilic esophagitis. Nat. Med. 19:1005–1013. 23872715 10.1038/nm.3281 PMC3951204

[phy214261-bib-0019] Radano, M. C. , Q. Yuan , A. Katz , J. T. Fleming , S. Kubala , W. Shreffler , et al. 2014 Cesarean section and antibiotic use found to be associated with eosinophilic esophagitis. J. Allergy Clin. Immunol. Pract. 2: 475–477.e1. 25017541 10.1016/j.jaip.2014.02.018

[phy214261-bib-0020] Sherrill, J. D. , and M. E. Rothenberg . 2011 Genetic dissection of eosinophilic esophagitis provides insight into disease pathogenesis and treatment strategies. J Allergy Clin Immunol 128:23–32; quiz 33–24. 21570716 10.1016/j.jaci.2011.03.046 PMC3129465

[phy214261-bib-0021] Sherrill, J. D. , K. Kc , D. Wu , Z. Djukic , J. M. Caldwell , E. M. Stucke , et al. 2014 Desmoglein‐1 regulates esophageal epithelial barrier function and immune responses in eosinophilic esophagitis. Mucosal. Immunol. 7:718–729. 24220297 10.1038/mi.2013.90 PMC3999291

[phy214261-bib-0022] Stefka, A. T. , T. Feehley , P. Tripathi , J. Qiu , K. McCoy , S. K. Mazmanian , et al. 2014 Commensal bacteria protect against food allergen sensitization. Proc. Natl. Acad. Sci. USA 111:13145–13150. 25157157 10.1073/pnas.1412008111 PMC4246970

[phy214261-bib-0023] Warners, M. J. , B. J. Vlieg‐Boerstra , J. Verheij , B. D. van Rhijn , M. T. Van Ampting , L. F. Harthoorn , et al. 2017 Elemental diet decreases inflammation and improves symptoms in adult eosinophilic oesophagitis patients. Aliment Pharmacol. Ther. 45:777–787. 28112427 10.1111/apt.13953 PMC5324627

[phy214261-bib-0024] Zimmermann, P. , N. Messina , W. W. Mohn , B. B. Finlay , and N. Curtis . 2019 Association between the intestinal microbiota and allergic sensitization, eczema, and asthma: a systematic review. J. Allergy Clin. Immunol. 143:467–485. 30600099 10.1016/j.jaci.2018.09.025

