# Supplementary material for: A decreased abundance of clostridia characterizes the gut microbiota in eosinophilic esophagitis
Source: Physiol Rep. 2019 Oct 24;7(20):e14261. doi: 10.14814/phy2.14261 (PMC6813259; doi:10.14814/phy2.14261)

[
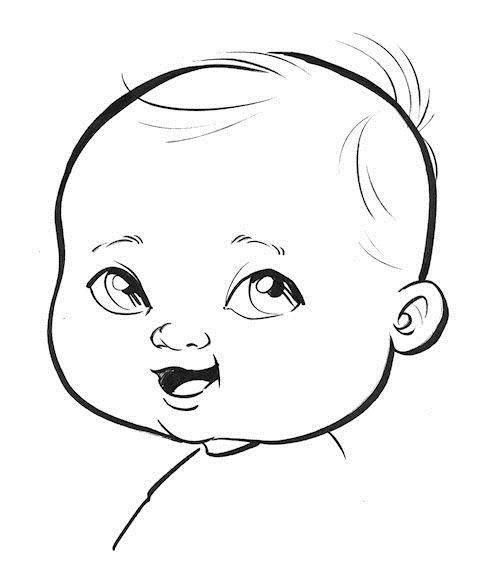
](https://www.google.com/url?sa=i&rct=j&q=&esrc=s&source=images&cd=&ved=2ahUKEwiCnJG4r5fjAhUFHjQIHc0IDpcQjRx6BAgBEAU&url=https%3A%2F%2Fwww.pinterest.com%2Fpin%2F6966574395619021%2F&psig=AOvVaw2-eFJ3fEQ_enfA83-d7SRX&ust=1562195917956501)

Caesarian Section

Antibiotic Exposure

Lack of Breast Feeding

Allergic March?


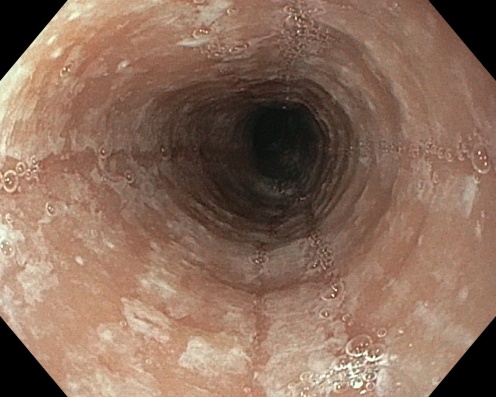

Supplement: Supplementary file 1 — Figure S1: Proposed pathogenesis of microbiome in eosinophilic esophagitis. [file PHY2-7-e14261-s001.docx]
